# Genetic diversity of *Ancylostoma ceylanicum* and first molecular detection of *Ancylostoma braziliense* in stray dogs from Sarawak, Malaysia

**DOI:** 10.1038/s41598-025-99092-8

**Published:** 2025-04-26

**Authors:** Ahmad Syatir Tahar, Sultana Parvin Habeebur-Rahman, Khatijah Yaman, Romano Ngui, Li Li Andrea Lim, Josprin Edwin, Nur Suhada Saboden, Tracy Manggi, Cheng Siang Tan

**Affiliations:** 1 Centre for Tropical and Emerging Diseases, Faculty of Medicine and Health Sciences, Universiti Malaysia Sarawak, Kota Samarahan, Sarawak Malaysia; 2 Department of Paraclinical Sciences, Faculty of Medicine and Health Sciences, Universiti Malaysia Sarawak, Kota Samarahan, Sarawak Malaysia; 3 Department of Veterinary Services Sarawak, Lot 877 Jalan Semenggok, Off Batu 12, Jalan Kuching-Serian, 93250 Kuching, Sarawak Malaysia

**Keywords:** Ancylostomiasis, Neglected tropical diseases, Molecular ecology, Evolutionary analysis, Molecular epidemiology, One health, Parasitology, Phylogenetics

## Abstract

Hookworms are blood-sucking intestinal parasites that can cause anaemia and protein loss in humans. *Ancylostoma ceylanicum,* a zoonotic hookworm species of dogs, is the second most common cause of human hookworm infections. With the increasing anthelmintic resistance risks and the uncontrolled stray dog population in Sarawak Borneo, East Malaysia, understanding the genetic structure of *A. ceylanicum* is crucial for tracking mutation patterns and assessing zoonotic transmission risks. This study determined the prevalence and genetic diversity of dog hookworm species using microscopy, PCR and sequencing, revealing *A. ceylanicum* (43.6%; 89/204), followed by mixed infections of *A. ceylanicum* and *A. braziliense* (9.3%; 19/204), single infections of *A. caninum* (6.3%; 13/204), and *A. braziliense* (1.4%; 3/204) in stray dogs in East Malaysia (Sarawak Borneo). Phylogenetic analysis of the cytochrome oxidase subunit 1 (COX1) gene showed that *A. ceylanicum* from Sarawak Borneo clustered across all major clades, indicating high genetic divergence and admixture. Haplotype analysis revealed that the Malaysian *A. ceylanicum* population highly mirrors those in Cambodia and Thailand, suggesting significant gene flow across Southeast Asia, while regional disparities exist compared to other countries. These findings provide critical epidemiological insights for hookworm control strategies, including stray dog management and potential adjustments to mass drug administration programs. The high genetic connectivity of *A. ceylanicum* population across borders underscores the need for enhanced surveillance, One Health approaches, and monitoring anthelminthic resistance to mitigate the risk of zoonotic transmission.

**Supplementary Information:**

The online version contains supplementary material available at 10.1038/s41598-025-99092-8.

## Introduction

Hookworms are parasitic roundworms of the Family Ancylostomatidae that infect humans and animals. As one of the soil-transmitted helminths (STHs), hookworm infection is among the most debilitating neglected tropical diseases (NTDs) that significantly impact health and socioeconomic status. Despite implicating 4 million disability-adjusted life years (DALYs) and productivity losses of up to $139 billion per year^1^, hookworm infections remain underestimated. *Ancylostoma duodenale* and *Necator americanus* were classically considered the most important species causing human infections, but molecular studies have identified *A. ceylanicum* as the second most common. Among the five dog hookworm species that infect humans, *A. caninum* causes eosinophilic enteritis and cutaneous larva migrans (CLM); *A. braziliense* is the main cause of intense itching and prolonged creeping eruption due to CLM; and *Uncinaria stenocephala* causes rare CLM and mild gastrointestinal symptoms. Notably, *A. ceylanicum* is the only species that can complete the life cycle in humans^2,3^, causing natural patent infection and eosinophilic enteritis^2,3^, which can result in iron-deficiency anaemia and protein loss due to its blood-sucking behaviour^4^.

Stray dogs play an important role in bridging the sylvatic and domestic cycle of diseases, as observed in local rabies outbreaks in Sarawak. Their overpopulation—driven by uncontrolled breeding, abandonment, urbanisation, and the absence of predators—complicates efforts to control zoonotic hookworm transmission. Free-ranging stray dogs defaecate in open areas, facilitating hookworm transmission to pet dogs and humans. In the host’s small intestine, adult hookworms mate and lay eggs, which are excreted in the faeces. Under favourable soil conditions, the eggs embryonate and hatch into non-infective, free-living rhabditiform larvae (L1), which develop into infective filariform larvae (L3), continuing the infection cycle. Humans become infected mainly through skin penetration by L3 larvae or, less commonly, by ingesting contaminated fruits and vegetables^5^.

Classical hookworm identification relied on morphometric analyses of eggs and adult worms, a diagnostic gold standard due to its simplicity and low cost. However, certain hookworm species are morphologically cryptic, which creates a bias in species identification^6–8^. For example, *A. ceylanicum* and *A. braziliense* were once considered a single species^9^ until a later discovery detailing the differentiation of the adult male worms of both species^8,10^. Other than that, *A. ceylanicum* was historically misclassified as *A. duodenale* based on egg morphology in human infection surveys, leading to underreported prevalence^11^. Considering these scenarios, morphometric analysis alone may be insufficient in epidemiological surveillance and may overlook mixed infections.

Dog hookworms have been well-described in Peninsular Malaysia^12–14^, with studies identifying *A. ceylanicum*, *A. caninum*, and *U. stenocephala*. However, no similar reports exist for Sarawak and other regions of Borneo. Previously, hookworms collectively accounted for 3 to 47.2% of human infections in Sarawak^15^, however, the surveillance lacked molecular data to distinguish the human and zoonotic hookworm species. This gap has hindered recognition of the role of animal control in managing hookworm infections. The geographical distinctiveness of East Malaysia (Borneo) may contribute to unique haplotypes compared to West Malaysia and other countries, warranting further study.

The first and second internal transcribed spacers (ITS-1 and ITS-2) of ribosomal DNA are commonly used genetic marker for hookworm species identification, but their limited sequence variability makes them less effective for distinguishing hookworm isolates within the same species. Parasite population genetics are shaped by gene flow and host mobility, with the cytochrome c oxidase subunit 1 (COX1) gene serving as a key marker for studying population genetic structure and evolution due to its high mutation rate, conserved structure, lack of recombination, and maternal inheritance^16^. Understanding genetic structure provides insights into the pattern of gene flow and the size of population—which both are key parameters for predicting the spread of anthelminthic resistance and the decline or re-emergence of the parasite populations after drug interventions^17^. To stay abreast of the latest epidemiological patterns of zoonotic dog hookworms, this study sought to determine the prevalence of zoonotic hookworms in stray dogs in East Malaysia (Sarawak Borneo), and characterise and analyse the genetic structure of *A. ceylanicum* using COX1 sequences from Malaysia and other countries. This study was conducted to characterise zoonotic hookworm species in stray dogs in Sarawak, Malaysia, and to determine the evolutionary pattern of locally circulating *A. ceylanicum* compared to those reported globally.

**Table 1:** The composition of *A. ceylanicum, A. braziliense*, and *A. caninum* with sex and age group of the stray dogs as detected using PCR and nucleotide sequencing.

Dog characteristics	Single infections			Mixed infections
	*Ancylostoma ceylanicum*	*Ancylostoma braziliense*	*Ancylostoma caninum*	Mixed of *A. ceylanicum* and *A. braziliense*
**Sex**				
Male				
62.2% (61/98)	41.8% (41/98)	0% (0/98)	8.1% (8/98)	12.2% (12/98)
Female				
59.4% (63/106)	45.2% (48/106)	2.8% (3/106)	4.7% (5/106)	6.6% (7/106)
**Age group**				
Puppy				
50.9% (26/51)	35.2% (18/51)	3.9% (2/51)	1.9% (1/51)	9.8% (5/51)
Adult				
64% (98/153)	46.4% (71/153)	0.6% (1/153)	7.8% (12/153)	9.1% (14/153)
**Subtotal**	43.6% (89/204)	1.4% (3/204)	6.3% (13/204)	9.3% (19/204)
**Total (N:204)**	60.7% (124/204)			

“N” indicates the number of samples. The numbers in the parentheses indicate the number of positive samples out of the total number of samples based on sex and age groups.

## Materials and methods

### Ethical approval

All experimental procedures were approved by the Universiti Malaysia Sarawak Animal Ethics Committee (UNIMAS/AEC/R/F07/070) and conducted in accordance with the Animal Research: Reporting of In Vivo Experiments (ARRIVE) guidelines and relevant local regulations.

### Study site and collection of dog faecal samples

Sarawak is located on Borneo Island in East Malaysia and, along with West Malaysia (Peninsular Malaysia), collectively forms the Federation of Malaysia. Presently, Sarawak is amid widespread rabies outbreaks, which points to the crisis of stray dog overpopulation and its role in zoonotic disease transmission. This study involved 204 stray dogs (153 adults and 51 puppies, comprising 98 male and 106 female dogs) captured by the local municipalities in collaboration with the Department of Veterinary Services (DVS) in Sarawak. Fresh faecal samples were collected directly from the rectum of each dog and transported to the Medical Microbiology Laboratory, Universiti Malaysia Sarawak, for analysis.

### Microscopic examination of hookworm eggs

Faecal samples were preserved in 2.5% potassium dichromate and stored at 4 °C. The samples were subjected to the formalin-ether sedimentation method^18^, and the final sediment was stained with 0.85% iodine solution. Hookworm eggs were identified under a light microscope at 100× and 400× magnifications.

### Molecular characterisation of dog hookworm species

Genomic DNA was extracted from faecal samples using the High Pure Viral Nucleic Acids (Roche, Switzerland), with an extended Proteinase K incubation at 72 °C for 2 h. Two PCR assays targeting the ITS1, 5.8S, and ITS2 regions of zoonotic hookworm species were conducted. The first assay used the primers RTGHF1 (5′-CGT GCT AGT CTT CAG GAC TTT G-3′) and RTABCR1 (5′-CGG GAA TTG CTA TAA GCA AGT GC-3′), which amplify a 545-bp fragment to detect *A. caninum*, *A. ceylanicum*, and *U. stenocephala*. The second assay used the primers RTGHF1 and RTAYR1 (5′-CTG CTG AAA AGT CCT CAA GTC C-3′), which amplify a 673-bp fragment specifically to detect *A. braziliense*^11^. Both PCR assays were performed separately, each in a final volume of 50 µl containing 1 × *Taq* buffer with (NH_4_)_2_SO_4_ (Thermo Fisher Scientific), 2.5 mM MgCl_2_ (Thermo Fisher Scientific)_,_ 0.2 mM dNTP mix (Thermo Fisher Scientific), 0.4 µM of each primer, 0.0125 U *Taq* DNA Polymerase, recombinant (Thermo Fisher Scientific) and 2 µl of DNA template. Amplification reactions were performed in a Mastercycler® X50 PCR Thermocycler (Eppendorf, Germany). PCR thermocycling conditions were: initial denaturation at 95 °C for 5 min, 40 cycles each of denaturation at 95 °C for 30 s, annealing at 60 °C for 30 s, elongation at 72 °C for 50 s, and final elongation at 72 °C for 5 min. Amplicons were sequenced using ABI® BigDye Terminator Cycle Sequencing Kit v3.1 and performed in the ABI PRISM 3730xl Genetic Analyser (Applied Biosystems, USA). Sequences of the ITS1, 5.8S, and ITS2 regions were deposited in GenBank (Supplementary Dataset File 1).

### Phylogenetic and haplotype analysis of *Ancylostoma ceylanicum*

Samples confirmed as *A. ceylanicum* by PCR and sequencing underwent haplotype analysis. Briefly, PCR was performed to amplify a 377-bp fragment of the COX1 gene specific to *A. ceylanicum* using AceyCOX1F (5′-GCT TTT GGT ATT GTA AGA CAG-3′) and AceyCOX1R (5′-CTA ACA ACA TAA TAA GTA TCA TG-3′)^19^ with *Taq* DNA Polymerase, recombinant (Thermo Fisher Scientific). The final volume and concentrations of each PCR component in this PCR assay were similar to the above. Thermocycling conditions were: initial denaturation at 95 °C for 5 min, 40 cycles each of denaturation at 95 °C for 30 s, annealing at 55 °C for 30 s, elongation at 72 °C for 50 s, and final elongation at 72 °C for 5 min. Amplicons were sequenced as described above. The phylogenetic tree was constructed using MEGA software version 11 (https://www.megasoftware.net/), and haplotype analysis was performed using DnaSP version 6 program (http://www.ub.edu/dnasp)^20^. To determine *A. ceylanicum* gene flow, we retrieved and included global sequences deposited in Genbank (https://www.ncbi.nlm.nih.gov/genbank/about/) in the analysis. Median-Joining Network analysis was conducted using DnaSP version 6 and PopArt software version 1.7.2 (https://popart.maths.otago.ac.nz/)^21^. Sequences of the COX1 gene were deposited in GenBank (Supplementary Dataset File 1).

## Results

### Prevalence of hookworm species

A total of 60.7% (124/204) of the dogs were positive with at least one hookworm species by PCR and nucleotide sequencing, comprising single infections with *Ancylostoma ceylanicum* (43.6%; 89/204) presenting the most prevalent hookworm species, followed by mixed infections with *A. ceylanicum* and *A. braziliense* (9.3%; 19/204), single infections with *A. caninum* (6.3%; 13/204), and *A. braziliense* (1.4%; 3/204) (Table 1; Supplementary Figure 1). In contrast, 42.6% (87/204) of the faecal samples were positive with hookworm eggs via microscopy (Supplementary Dataset File 2; Supplementary Figure 2). The breakdown of the characteristic data of the dogs indicates that male (62.2%) versus female (59.4%) and puppies (50.9%) versus adult dogs (64%) had a relatively similar positivity rate.

### Phylogenetic analysis of *A. ceylanicum*

Analysis of the COX1 gene sequences of *A. ceylanicum* from Malaysia and other countries categorised the species into five phylogenetic clades (Clade A-E). Isolates from this study were distributed across all clades, including those clustering with isolates that had previously infected humans, dogs, cats, and coyotes. Notably, three isolates formed a unique clade with an *A. ceylanicum* strain that previously caused a human infection in Australia (AJ407937), supported by an 89% bootstrap value.

**Fig. 1:**
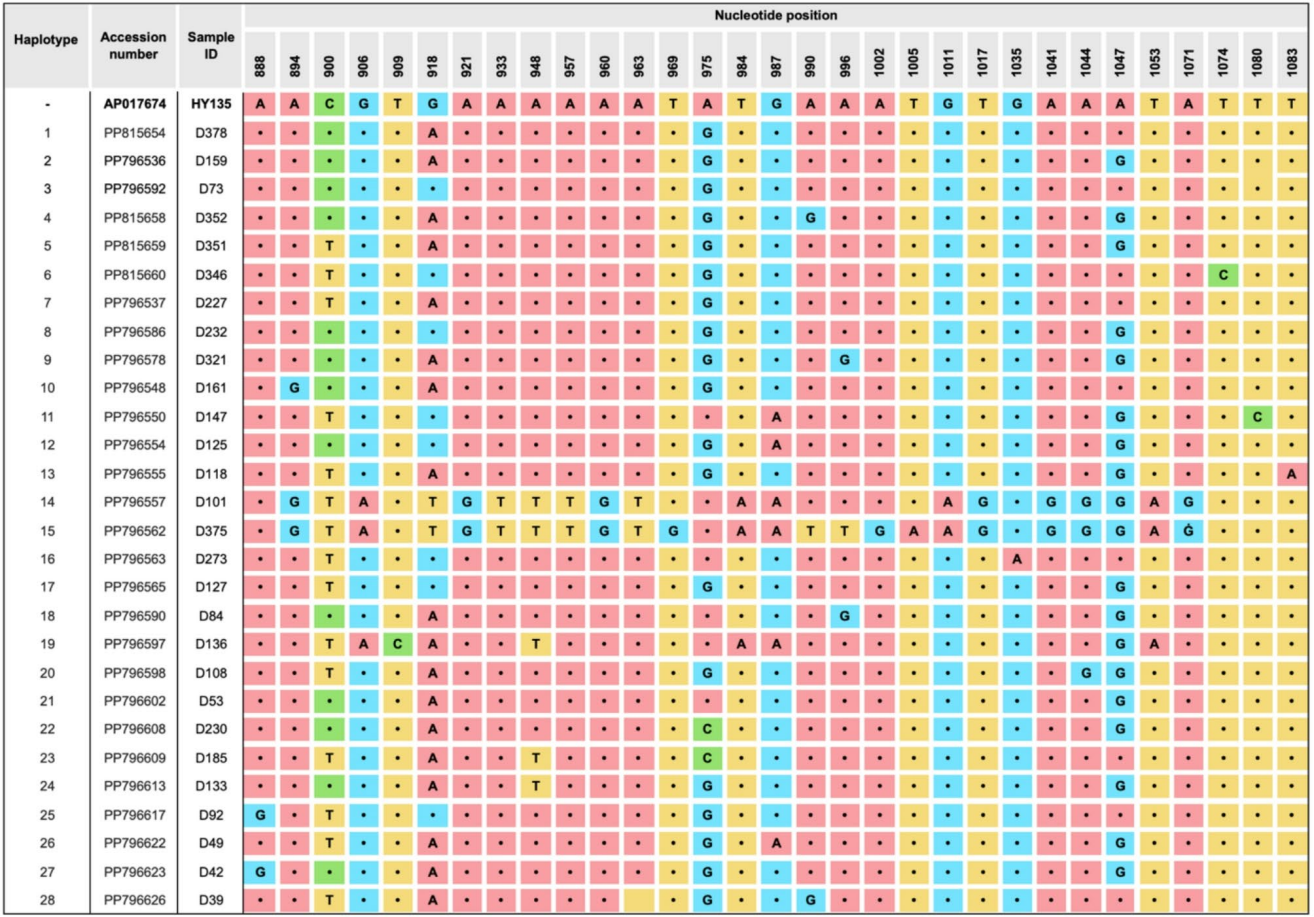
Nucleotide variations across the COX1 gene haplotypes of *Ancylostoma ceylanicum* this study versus the COX1 gene sequence of *A. ceylanicum* HY135 (accession number: AP017674) as the reference sequence, which was also used to obtain the nucleotide positions. There are 28 haplotypes for *A. ceylanicum* in East Malaysia (Borneo Malaysia). High nucleotide substitutions were observed for the positions of 900, 918, 975, and 1047. Note: Each haplotype may contain several similar isolates, thus only one representative was included for each haplotype. A indicates adenine, T indicates thymine, G indicates guanine, C indicates cytosine, and • indicates consensus nucleotide base with the reference sequence.

There were 28 haplotypes of *A. ceylanicum* identified in this study (Fig. 1), with high nucleotide substitutions were observed at positions 900, 918, 975, and 1047, with the highest at position 975 (adenine to guanine/cysteine). Other nucleotide positions (88–1083) showed relatively low levels of substitution.

### Global population analysis of *A. ceylanicum*

**Fig. 2:**
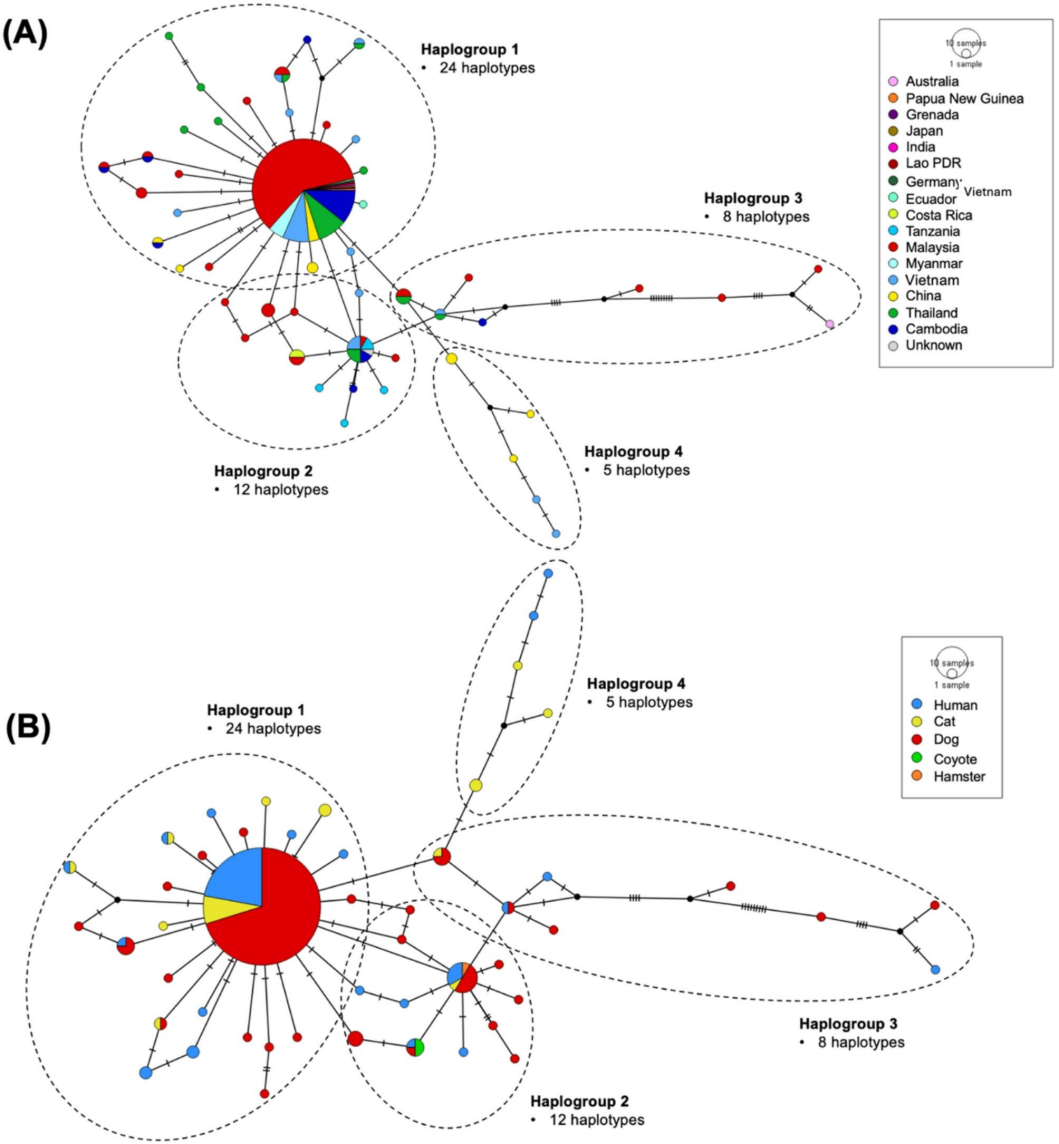
Population analyses using median-joining network show the evolutionary pattern of *Ancylostoma ceylanicum* haplotypes across the world. (**A**) The haplotype distribution network based on country category, (**B**) The haplotype distribution network based on host category. The size of the circle corresponds to the number of *A. ceylanicum* individuals in that population, whereas the number of hash marks on the connecting line between two populations indicates the number of nucleotide variations. Haplotypes are indicated with the colored circles, whereas haplogroups are marked with black dashed circles.

To investigate the haplotype clustering of *A. ceylanicum* based on geographic (country) and host distribution, we performed population analyses to understand the haplotype patterns and gene flows (Fig. 2). The population analysis by geographic distribution categorised *A. ceylanicum* haplotypes into four haplogroups (Haplogroup 1–4), comprising 49 haplotypes. Haplogroup 1 was the largest haplogroup (indicated by the largest circle), indicating a focal concentration of the parasite individuals. The highest number of isolates originated from Malaysia (142), followed by Thailand (30), Vietnam (28), Cambodia (27), China (14), Tanzania (5), Costa Rica (2), Ecuador (2), Japan (1), Germany (1), Grenada (1), Papua New Guinea (1), Lao PDR (1) and India (1) (Fig. 2A).

The population analysis by host distribution categorised *A. ceylanicum* haplotypes into four haplogroups (Haplogroup 1–4). Haplogroup 1 was the largest haplogroup (indicated by the largest circle), indicating a focal concentration of the parasite individuals. The highest number of isolates was hosted by dogs (173), followed by humans (64), cats (27), coyotes (2), and hamster (1) (Fig. 2B).

**Fig. 3:**
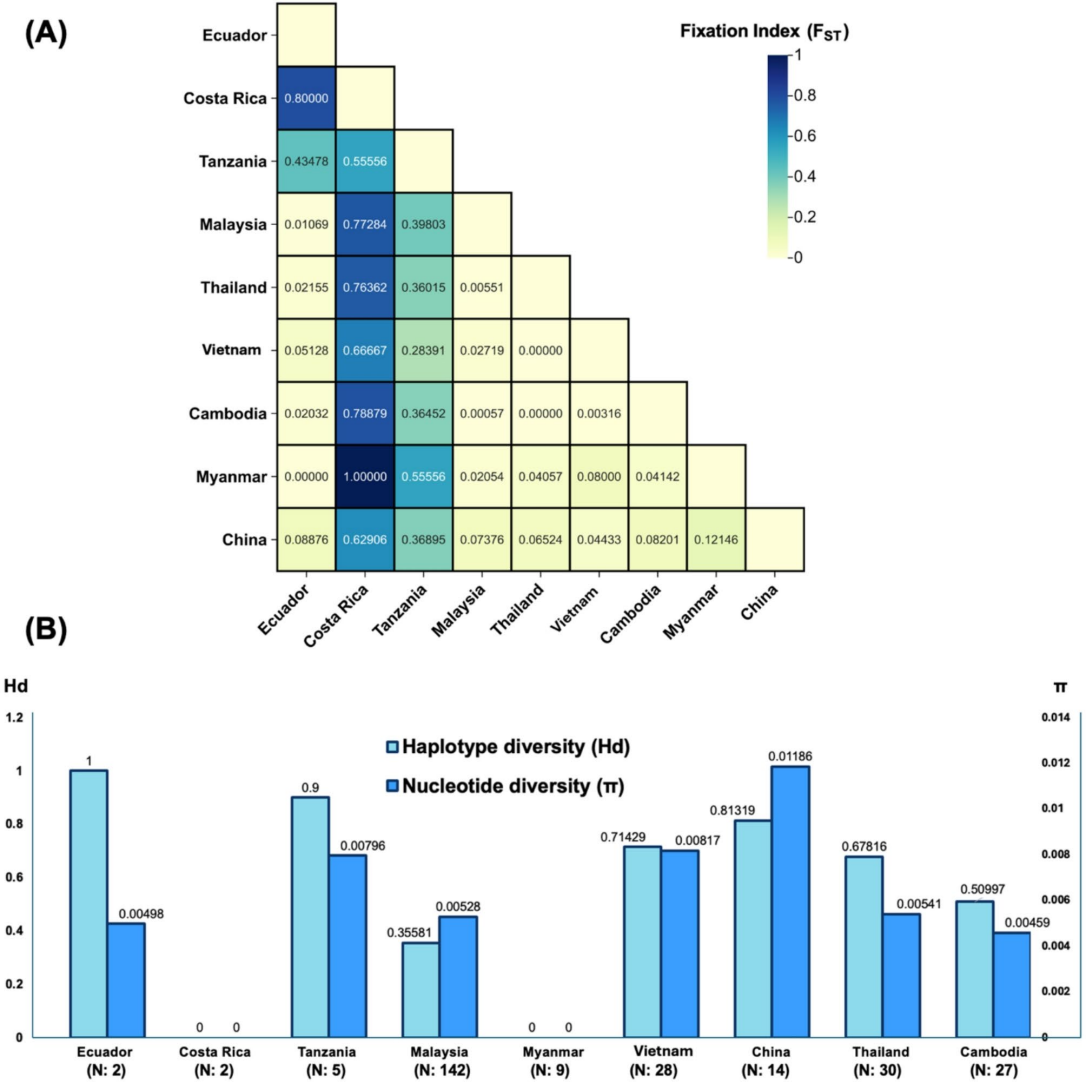
(**A**) Pairwise matrix for genetic divergence of *Ancylostoma ceylanicum* population across the world involving nine countries (Ecuador, Costa Rica, Tanzania, Malaysia, Thailand, Vietnam, Cambodia, Myanmar, and China). “F_ST_” indicates “fixation index”. The fixation index represents the total genetic variation of a population relative to genetic variation in all populations, whereas a low F_ST_ value indicates a low degree of genetic divergence between the populations. Note: Countries with only one *A. ceylanicum* sequence available in the GenBank, such as Australia, Japan, Lao PDR, and Germany, were excluded from this population analysis. Among the nine countries, Cambodia showed the lowest F_ST_ value, while Costa Rica showed the highest in comparison to Malaysia (**B**) The haplotype diversity (Hd) and nucleotide diversity (π) of *A. ceylanicum* based on countries. “N” indicates the total COX1 sequences from the respective country used in this analysis. All F_ST_, Hd, and π values were statistically significant (*P* < 0.001).

Pairwise comparison of COX1 sequences of *A. ceylanicum* isolates from Malaysia versus other countries revealed varying degrees of genetic divergence (Fig. 3). The least divergent population from Malaysia was Cambodia (F_ST_: 0), followed by Thailand (F_ST_: 0.005), Ecuador (F_ST_: 0.01165), Myanmar (F_ST_: 0.02206), Vietnam (F_ST_: 0.02586). In contrast, China (F_ST_: 0.08116), Tanzania (F_ST_: 0.39373), and Costa Rica (F_ST_: 0.76641) showed greater divergence from the Malaysian population (Fig. 3A). Haplotype and nucleotide diversity varied across regions (Fig. 3B). The highest diversity was observed in China (Hd: 0.81319; π: 0.01186), followed by Tanzania (Hd: 0.9; π: 0.00796), Ecuador (Hd: 1; π: 0.00498), Vietnam (Hd: 0.71429; π: 0.00817), Thailand (Hd: 0.67816; π: 0.00541), and Cambodia (Hd: 0.50997; π: 0.00459). Malaysia exhibited moderate diversity (Hd: 0.35581; π: 0.00528), whilst Myanmar and Costa Rica exhibited no diversity (Hd: 0; π: 0).

## Discussion

We identified *Ancylostoma ceylanicum* as the most prevalent hookworm species (43.6%), followed by mixed infections with *A. ceylanicum* and *A. braziliense* (9.3%), single infections of *A. caninum* (6.3%) and *A. braziliense* (1.4%). Historically, the distribution of *A. braziliense* was known to be restricted to the Americas, particularly the warm southern United States, southeastern U.S. (e.g. Gulf Coast states), Central and South America, and the Caribbean^22^. But, as the understanding of epidemiology improved, additional cases were reported in the Africa^23,24^, Asia (e.g. Southern and Southeast Asia)^6,25^ and Oceania (e.g. Northen Australia)^6^. This distribution pattern indicates that *A. braziliense* thrives in tropical and subtropical regions worldwide.

Presently, confirmation of *A. braziliense* has been reported only sporadically in Southeast Asia (SEA), and its true prevalence remains unclear. CLM is a clinical manifestation of several hookworm species. Although not all cases of CLM can be attributed to *A. braziliense,* the consistently high regional incidence involving residents^26–28^ and returning travellers from the SEA countries^29–32^ indirectly suggests the presence of *A. braziliense* as a significant causative agent. The larvae of *A. braziliense* mainly cause intense itching and prolonged creeping eruption, characterised by long, serpiginous tracks, compared to the pruritic papular lesions produced by the larvae of *A. ceylanicum* and *A. caninum*^33^.

In Malaysia, *A. braziliense* was first detected in a cat in 2012 (2%)^25^ as confirmed by molecular tools. Later, the species remained undetected, marking this as the first molecular confirmation of *A. braziliense* in dogs in Malaysia. The prevalence of *A. braziliense* was remarkably low in dog hosts in the SEA, despite the correct PCR primers being utilised to detect the species. Molecular detection in cats has been reported in Malaysia^25^ and Indonesia^34^, but this study provides only the second confirmed case of *A. braziliense* in dogs in the SEA after Cambodia (20%)^35^. This finding suggests either separate lineages of *A. braziliense* in dogs versus cats or that the true prevalence is rather low.

PCR detection showed a higher detection rate (60.7%; 124/204) compared to microscopy (43%; 87/204) (Supplementary Dataset File 2), indicating that relying solely on microscopy underestimates the true prevalence of hookworms, especially in low-intensity infections. Relying solely on microscopy to monitor human infections can misidentify hookworm eggs as those of morphologically similar strongylid nematodes, such as *Oesophagostomum bifurcum*, *Trichostrongylus* spp., and *Ternidens* sp.^36–38^*,* especially in areas these parasites co-circulate.

Sex and age groups of the dogs were independent of the infection risk, with the exception of female adult dogs that are nursing puppies and can spread the infections via the transmammary route. Transmammary infection occurs when arrested tissue-stage larvae of the bitches are reactivated, migrate to the mammary glands, and are then passed in the colostrum and milk. To date, no clinical data has specifically proved the role of *A. ceylanicum* and *A. braziliense* in this context, but *A. caninum* is well known for that transmission^39–41^.

**Fig. 4:**
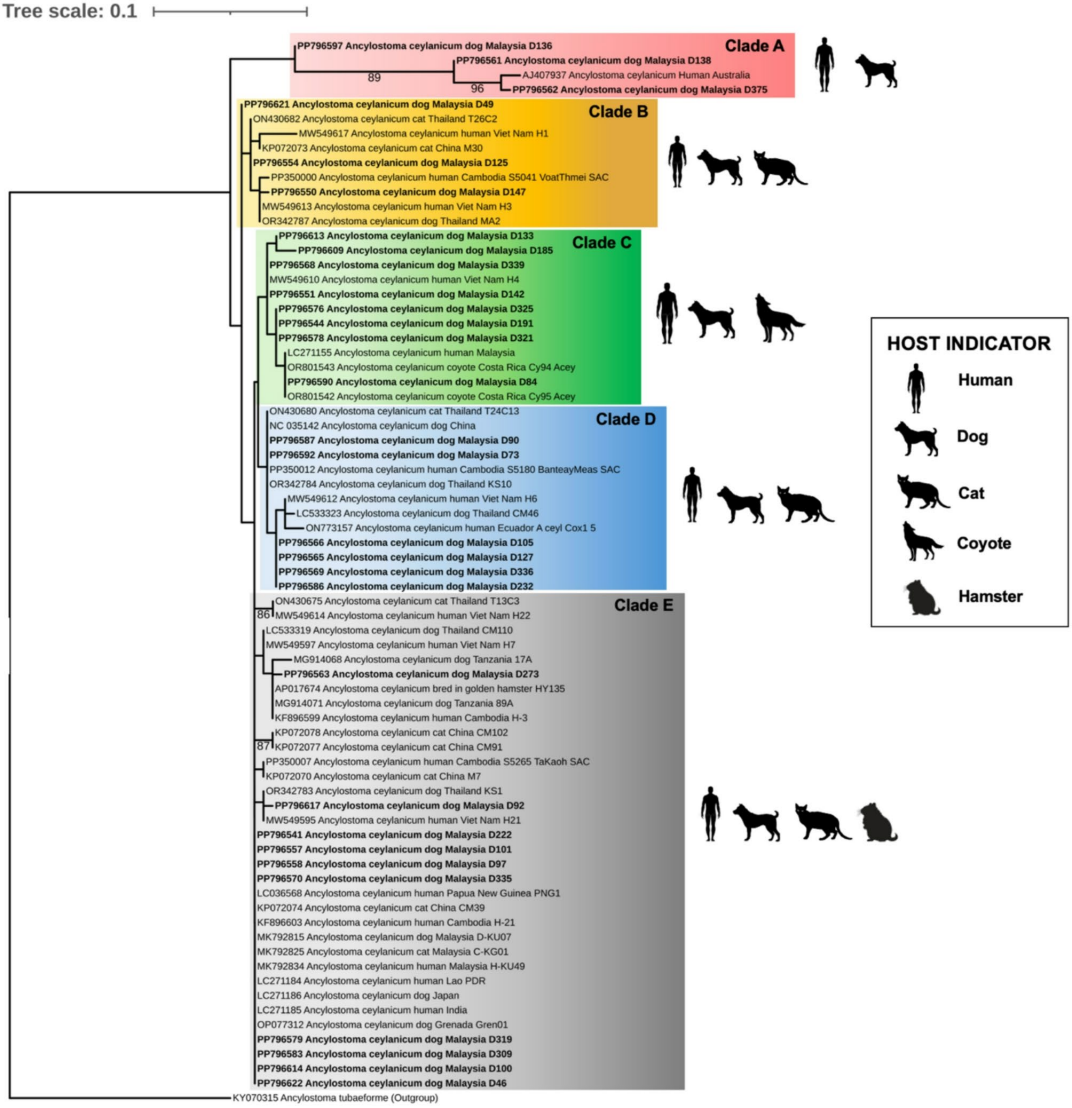
Phylogenetic analysis of COX1 gene shows *Ancylostoma ceylanicum* were categorized into five clades (**A**–**E**). *A. ceylanicum* isolates from this study are indicated in boldface. The bootstrap values are indicated at the branch nodes, and the individual clade is considered well-supported if the bootstrap value is at least 70%. *A. tubaeforme* (accession number: KY070315) was used as an outgroup.

*A. ceylanicum* in Sarawak (East Malaysia) formed five phylogenetic clades (Clade A–E) (Fig. 4), highlighting the highly admixed gene pool relative to the global isolates. Some clades consisted of multiple host species, confirming cross-species transmission beyond dogs and humans. Notably, three local isolates formed a distinct clade with an Australian isolate linked to human infection (Clade A), indicating the significant substructuring compared to other countries. Haplotype network analysis revealed a dynamic population structure, likely shaped by phylogeography or parasite-host adaptation, with a focal expansion in Haplogroup 1 (Fig. 2) suggests possible host mobility and parasite-host adaptation in the hookworm transmission^42^.

A previous study reported moderate genetic divergence between Malaysian *A. ceylanicum* and those from Cambodia (F_ST_: 0.0999) and Thailand (F_ST_: 0.1526)^43^. However, this conclusion was drawn based only on the isolates restricted to West Malaysia (Peninsular Malaysia), therefore the broader *A. ceylanicum* population in Malaysia was not adequately covered. In our current study, we supplemented the database with the latest COX1 sequences in East Malaysia (Sarawak Borneo), refining the conclusion that the populations in these three countries share a high degree of genetic similarity (F_ST_: 0.00057 and 0.00551) (Fig. 3A). This panmictic population structure suggests historical gene flow events. This finding shows that a highly similar population of *A. ceylanicum* circulate in Malaysia, Cambodia, and Thailand, while regional genetic disparities exist in other countries. Parasite transmission is largely host-dependent due to limited independent mobility. This population structure is an indication of the parasite-host relationship since the population genetics of parasites must be structured to a certain extent to adapt to the hosts^42^ for completing the life cycle and facilitating the transmission.

The extensive admixture of *A. ceylanicum* in Malaysia is likely driven by host mobility (such as humans, dogs, or cats), arising naturally or through anthropogenic factors. Pairwise comparisons (Fig. 3A) showed that genetic divergence increased with geographic distance from Malaysia. Hookworms can spread globally via travelers^44^, deployed military personnel, migrant workers^45^, humanitarian assistance, and animal trade, particularly within SEA. Moreover, other factors influencing gene flow include topography, parasite population size, and host mixing dynamics^46^. The substantial genetic diversity observed in this study raises concerns about the potential emergence of anthelminthic-resistant haplotypes since resistance emerges randomly after anthelminthic exposure. Similar patterns of genetic adaptation and transcriptomic plasticity have been observed in *Haemonchus contortus*, a ruminant parasite that has developed benzimidazole resistance^47^. The emergence of drug resistance typically involves rare resistance alleles occurring with a low frequency. This event is influenced by several factors, including population size, diversity, mutation rate of relevant gene(s), and the relative fitness of the mutated compared to the wild-type parasite^48^. Thus, the genetic diversity and admixture presented in this study underscore the need for continuous surveillance to monitor the potential emergence of resistant *A. ceylanicum*.

## Conclusion

The findings of this study provide critical epidemiological insights for hookworm control strategies, including stray dog management and potential adjustments to mass drug administration programs. The high genetic similarity of *A. ceylanicum* population across borders underscores the need for enhanced surveillance, One Health approaches, and monitoring of anthelminthic resistance to mitigate the risk of zoonotic transmission. The high prevalence of *A. ceylanicum* in stray dogs indicates the presence of public health risk, although the zoonotic potential of this hookworm is often underestimated particularly in the context of animal control.

## Electronic supplementary material

Below is the link to the electronic supplementary material.

Supplementary Material 1

Supplementary Material 2

Supplementary Material 3

## Data Availability

All data generated or analysed during this study are included in the Supplementary Information files.
