# Supplementary material for: Genetic diversity of Ancylostoma ceylanicum and first molecular detection of Ancylostoma braziliense in stray dogs from Sarawak, Malaysia
Source: Sci Rep. 2025 Apr 26;15:14586. doi: 10.1038/s41598-025-99092-8 (PMC12032019; doi:10.1038/s41598-025-99092-8)

**Supplementary information: Figure 1**

Representative of agarose gel image for cytochrome b subunit 1 gene (COX1) of *Ancylostoma ceylanicum*

**
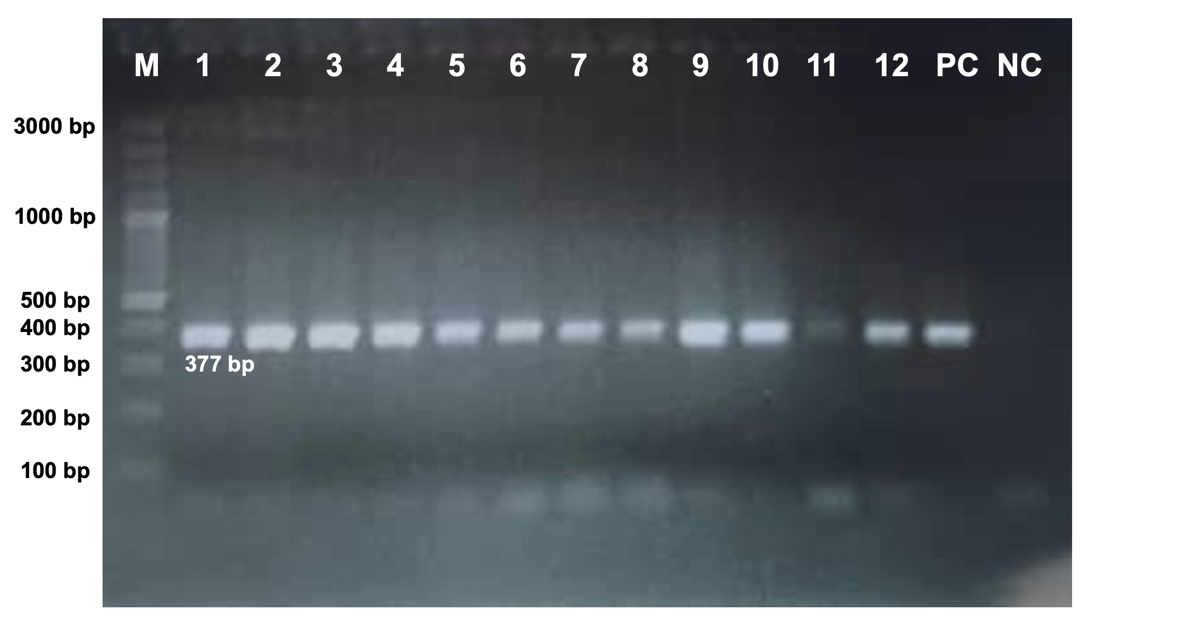
**

PCR amplification of the COX1 gene of *A. ceylanicum,* producing 377-bp PCR bands. M: 100-bp plus molecular weight ladder (Thermo Scientific, USA), 1-15 indicates samples on this study, 1: D332, 2: D335, 3: D336, 4: D339, 5: D346, 6: D351, 7: D352, 8: D353, 9: D357, 10: DD359, 11: DD375, 12: D378, PC: positive control, NC: negative control.

**Supplementary information: Figure 2**

Representative images of the hookworm eggs were detected using microscopy at 400× magnification.


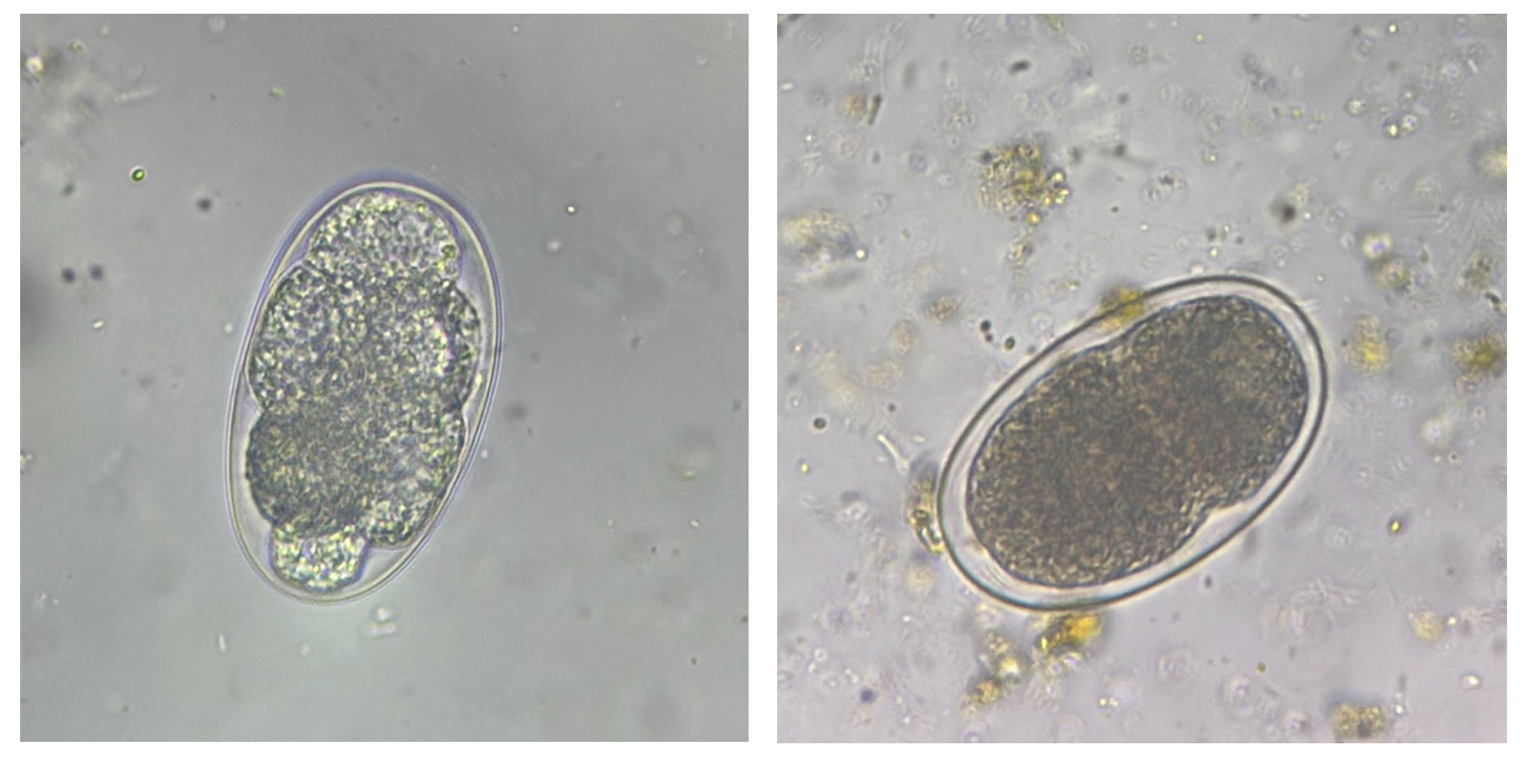

Supplement: Supplementary file 3 — Supplementary Material 3 [file 41598_2025_99092_MOESM3_ESM.docx]
